# Atomic Depth Image Transfer of Large-Area Optical Quartz Materials Based on Pulsed Ion Beam

**DOI:** 10.3390/mi15070914

**Published:** 2024-07-15

**Authors:** Shuyang Ran, Kefan Wen, Lingbo Xie, Xingyu Zhou, Ye Tian, Shuo Qiao, Feng Shi, Xing Peng

**Affiliations:** 1 College of Intelligent Science and Technology, National University of Defense Technology, Changsha 410073, China; ranshuyang@nudt.edu.cn (S.R.); wenkefanedu@nudt.edu.cn (K.W.); lingbotse@163.com (L.X.); zhouxingyu@nudt.edu.cn (X.Z.); tianye@nudt.edu.cn (Y.T.); sqiao525@163.com (S.Q.); shifeng@nudt.edu.cn (F.S.); 2 Hunan Provincial Key Laboratory of Ultra-Precision Machining Technology, Changsha 410073, China; 3 Laboratory of Science and Technology on Integrated Logistics Support, Changsha 410073, China

**Keywords:** optical material processing, pulsed ion beam, sub-nano precision manufacturing, atomic and near-atomic-scale fabrication, pattern transfer

## Abstract

The high-efficiency preparation of large-area microstructures of optical materials and precision graphic etching technology is one of the most important application directions in the atomic and near-atomic-scale manufacturing industry. Traditional focused ion beam (FIB) and reactive ion etching (RIE) methods have limitations in precision and efficiency, hindering their application in automated mass production. The pulsed ion beam (PIB) method addresses these issues by enhancing ion beam deflection to achieve high-resolution material removal on a macro scale, which can reach the equivalent removal resolution of 6.4 × 10^−4^ nm. Experiments were conducted on a quartz sample (10 × 10 × 1 mm) with a specific pattern mask using the custom PIB processing device. The surface morphology, etching depth, and roughness were measured post-process. The results demonstrated that precise control over cumulative sputtering time yielded well-defined patterns with expected average etching depths and surface roughness. This confirms the PIB technique’s potential for precise atomic depth image transfer and its suitability for industrial automation, offering a significant advancement in microfabrication technology.

## 1. Introduction

Atomic and Close-to-Atomic-Scale Manufacturing (ACSM) is an ultra-high-precision processing technology that utilizes direct energy interactions with material atoms to remove, transfer, and deposit materials at the atomic scale. ACSM’s ability to control and manipulate atoms is crucial for manufacturing high-precision components such as microchips and sensors. This technology can overcome the limitations of conventional manufacturing methods and lead the development of next-generation manufacturing technologies [1]. The efficient preparation of large-area microstructures is vital for the advancement of modern high-tech industries and science, making it a key growth factor for ACSM. This is one of the primary applications within the ACSM field. Current mainstream processing methods include focused ion beam (FIB) and reactive ion etching (RIE). These micromachining technologies have rapidly advanced in ACSM and are increasingly being utilized.

RIE excels in uniform etching depth, mask selectivity, size control, and processing efficiency, making it particularly suitable for automated mass production. Under specific operating conditions for the ion etching silicon nitride process—such as a constant flux of 50 sccm, another flux of 10 sccm, a chamber air pressure of 11 Pa, and an etching power of 250 W—the RIE process achieves an etching rate of 509 nm/min [2]. However, due to the limitations in processing depth and width inherent to widely used RIE techniques, coupled with the complexity of their respective process flows, the applicability of distinct RIE processes is constrained. Consequently, these methods exhibit certain limitations in the realm of atomic-scale image transfer [3].

Focused Ion Beam (FIB) micromachining technology allows for the direct formation of precision microstructures without the need for a mask. The morphology of the processed structures depends on key parameters of the FIB sputter etching process, such as ion energy, ion beam current size, residence time at individual pixel positions, and total processing time [4,5]. Although FIB theoretically achieves nanoscale precision, it is less efficient in preparing microstructures. For example, in 3D optical microstructure-focused ion beam milling, a grayscale map of 32 μm × 32 μm, a dwell time of 10 μs, and 3000 machining cycles require over 3 minutes to machine a single area. This is insufficient for transferring large-area graphics [6] and may not meet ACSM’s efficiency requirements. Pulsed Ion Beam (PIB) technology is a processing method that enhances ion beam technology’s precision and efficiency to address these issues. Zhou Guangqi et al. leveraged the capability of low-energy pulsed ion beams to finely modulate material ablation, introducing a novel pulsed ion beam machining technique. This method integrates pulse technology into the fabrication of ultra-precision optical elements via ion beam machining. Theoretical examinations of the pulsed removal function’s attributes were complemented by experimental validation. The experimental results and analysis indicate that PIB improves ion beam control and large-area material processing efficiency in micro-nanostructures [7].

This paper focuses on the sputtering removal law of materials by pulsed ion beam and explores the feasibility of the atomic depth image transfer method based on PIB for theoretical research and experimental validation. The paper presents the derivation and analysis of a theoretical model based on sub-nanometer precision modification theory. It determines the velocity distribution vector, duty cycle vector, and cumulative sputtering time corresponding to different machining depths. Quartz samples are sputtered with varying cumulative sputtering times in the corresponding areas using laboratory-developed PIB machining equipment IBF-700 [7]. This process etches the sample surfaces to target depths. Subsequently, a white light interferometer was used to measure and compute the surface topography, etching depth, and surface roughness parameters and features of the processed samples. This analysis evaluates the surface topography, etching depth, and etching uniformity of the processed areas from both qualitative and quantitative perspectives. Additionally, a multidimensional lateral comparison was conducted with existing etching methods for comprehensive evaluation.

This paper is divided into five sections. The first section discusses the theory of pulsed ion beam etching and the graphic transfer method. The second section details the experimental apparatus and methodology. The third section presents a qualitative and quantitative analysis of the experimental results and research findings. The fourth section comprehensively evaluates and analyzes the accuracy of the proposed model and the feasibility of the methodology through qualitative and quantitative analyses, as well as cross-sectional comparisons. The final section summarizes the work and presents the research conclusions.

## 2. Pulsed Ion Beam Etching Theory and Pattern Transfer Methods

### 2.1. Pulsed Ion Beam Etching Theory

Pulsed ion beam processing is a deterministic material removal technology used for materials with different parameters and molecular characteristics. It requires stable and controllable removal properties. To establish a stable removal function, the velocity distribution vector of the material to the target state is derived by determining the intrinsic parameters of the material and the target machining depth of a given machining area. Then, the processing time vector corresponding to the target etching area is calculated.

**Figure 1:**
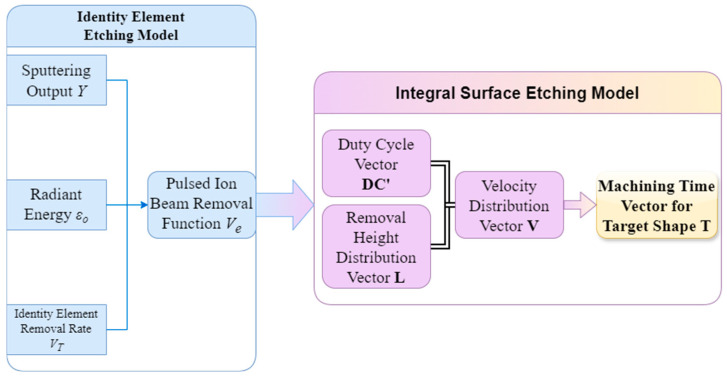
Model for pulsed ion beam etching.

This paper presents a model for etching the unit surface element, followed by an overall etching region model from point to surface. Finally, the target etching region processing time vector is derived from the expansion of the overall etching region etching model on the time scale. The comprehensive framework of model for pulsed ion beam etching is depicted in Figure 1.

#### 2.1.1. Modelling Unit Face Element Etching

The paper begins by establishing the unit surface element etching model. The empirical formula of Sigmund’s linear cascade collision theory [8,9], which has a lower time complexity than other major calculation methods, is used to calculate the sputtering yield Y.

The expression for the surface sputtering yield of an ion beam incident on an optical element is:

(1) $$Y(E)=\frac{3\alpha S_{n}(\varepsilon_{o})}{8\pi C_{0}U_{s}}$$

The value $C_{0}\approx 12\pi \times 0.219$ is determined by the Born–Mayer potential between low-energy atoms. $U_{s}$ is the surface binding energy per atom. The correction factor, α, is a function of the mass of the incident ion, $M_{1}$ (in Dalton, Da), and the atomic mass of the target material, $M_{2}$ (in Dalton, Da), calculated as $\alpha =0.15+\frac{0.13M_{2}}{M_{1}}$. The nuclear-stopping cross section, $S_{n}(\varepsilon_{o})$, is the induction coefficient, and $S_{n}^{TF}(\varepsilon )$ is the nuclear-stopping power based on the Thomas–Fermi potential. The formula is as follows:

(2) $$\left\{\begin{array}{l} S_{n}(\varepsilon_{o})=\frac{84.78Z_{1}Z_{2}}{{\left(Z_{1}{}^{\frac{2}{3}}+Z_{2}{}^{\frac{2}{3}}\right)}^{\frac{1}{2}}(M_{1}+M_{2})}\cdot S_{n}^{TF}(\varepsilon )\\ S_{n}^{TF}(\varepsilon )=\frac{3.441\varepsilon^{{}_{2}^{1}}\ln(\varepsilon +2.718)}{1+6.355\varepsilon^{\frac{1}{2}}+\varepsilon (6.882\varepsilon^{\frac{1}{2}}-1.708)} \end{array}\right.$$

where *Z*_1_ and *Z*_2_ are the atomic numbers of the incident ions and target atoms, respectively, and ε is the normalized energy. The calculation of ε is as follows:

(3) $$\varepsilon =\frac{0.0325M_{2}\varepsilon_{o}}{Z_{1}Z_{2}(M_{1}+M_{2}){\left(Z_{1}{}^{\frac{2}{3}}+Z_{2}{}^{\frac{2}{3}}\right)}^{\frac{1}{2}}}$$

where $\varepsilon_{o}$ is the incident ion energy.

From Equations (1)–(3), it can be seen that the sputtering yield Y is correlated with the energy of the incident ions deposited on the target material $\varepsilon_{o}$, and the trend for a particular material can be obtained by determining the parameters of the incident ions and the target material. Also, through the study of ion collision modeling by Sigmund et al., it can be found that the higher the energy deposited on the surface of the element, the higher the corresponding material removal efficiency, and the insufficient energy deposited on the surface of the element produces the phenomenon that the material atoms do not detach from the surface of the element.

**Figure 2:**
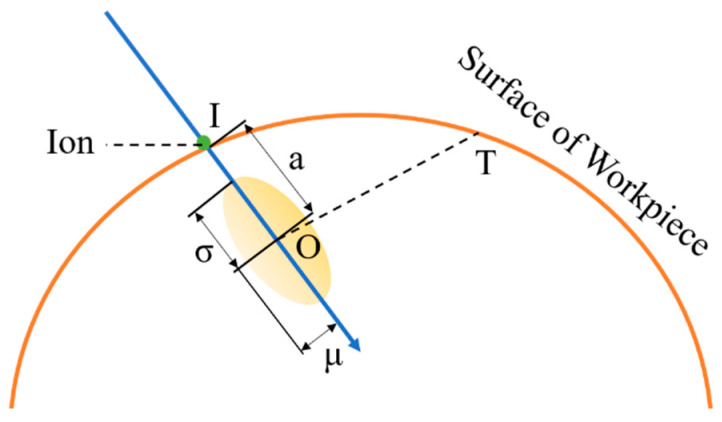
Incident ion energy deposition distribution.

As shown in Figure 2, according to the Sigmund sputtering theory, if the incident ion enters the surface of the element at point I and finally stops at point O, since the ion beam used by the pulsed ion beam adopts a focused ion source, the produced ion beam flux also essentially conforms to a Gaussian distribution. Therefore, the energy deposition can also be considered to exhibit a Gaussian distribution [10,11]. The energy deposited by the incident ions is centered at O within the material and has the following pattern:

(4) $$E(x,y,z)=\frac{\varepsilon_{o}e^{-\frac{z^{2}}{2\sigma^{2}}-\frac{x^{2}+y^{2}}{2\mu^{2}}}}{\sqrt{8\pi^{2}}\sigma \mu^{2}}$$

where *x*, *y*, and *z* denote the coordinates of the T-point in the reference coordinate system with O as the origin, *μ* denotes the scattering width of the incident ion energy perpendicular to the incidence direction, and *σ* denotes the scattering width parallel to the incidence direction.

In the process of ion beam treatment for the unit area, the ion beam flux is mainly introduced through the acceleration electric field between the screen grid and the acceleration grid. The voltages on the acceleration grid and the deceleration grid are usually kept constant, and the energy and quantity of extracted Ar+ ions can be changed by adjusting the size of the grid voltage. If the potential difference between the screen and the acceleration grid is reduced to 0 or a reverse electric field is formed, the acceleration electric field will not be able to extract Ar+ ions. The experimental processing procedure uses the laboratory-developed processing device IBF-700 based on the above principles and replaces the direct current source connected to the grid with a pulsed power source. This allows for the control of the grid voltage amplitude and duration by adjusting the duty cycle DC and frequency f (in hertz, Hz) [12].

The pulsed ion beam’s outgoing energy, $E_{o}$ (in electron volt, eV), is determined by f, DC, sputtering time t (in seconds, s), and the intensity of the ion beam I (in milliampere, mA) emanating from a single aperture. Under the two-thirds power law of single-aperture ion beams and the idealized two-level planar model [13], the current density of the single-aperture beam $J_{s}$ can be calculated via Equation (5):

(5) $$J_{s}=\frac{4}{9}\varepsilon_{0}\sqrt{\frac{2e}{M_{i}}}\frac{V^{\frac{3}{2}}}{L^{2}{}_{g}}$$

where V (in volt, V) signifies the total acceleration voltage, $L_{g}$ (in millimeter, mm) denotes the distance between the screen grid and the acceleration grid, $M_{i}$ (Da) stands for the mass of the ions, e denotes the charge of the electric charge, and $\varepsilon_{0}=8.854\times 10^{-12}F/m$, represents the electric constant.

Assuming that the beam current extracted through the gate aperture is uniform, the following can be deduced, the calculation $E_{o}$ is as follows:

(6) $$\left\{\begin{array}{l} E_{o}=\frac{DC\cdot t\cdot I}{f}\\ I=\frac{\pi d^{2}{}_{s}}{4}J_{s} \end{array}\right.$$

where $d_{s}$ (mm) refers to the diameter of the beam aperture.

The removal efficiency at point T can be expressed as determined in a previous laboratory study on ion beam removal efficiency [13]:

(7) $$V_{T}=\eta \int_{\Omega }DC\cdot J\cdot E_{o}d\Omega$$

dΩ is the unit grid cell of the workpiece, J is the beam density at point T, and η is the proportionality constant between the energy deposited on the surface of the workpiece after ion incidence and the removal rate of the material, which is related to the mass of the incident ions, the number of protons, and other parameters. The mathematical model for the removal function of the pulsed ion beam is expressed as follows:

(8) $$\left\{\begin{array}{l} V_{e}\approx \frac{\eta E_{o}DC\cdot J\cdot \cos\theta \cdot e^{-\frac{a^{2}\cos^{2}\theta }{2(\sigma^{2}\cos^{2}\theta +\mu^{2}\sin^{2}\theta )}}}{{\left(2\pi (\sigma^{2}\cos^{2}\theta +\mu^{2}\sin\theta )\right)}^{\frac{1}{2}}}\\ \eta =\frac{3}{4\pi^{2}nU_{b}C_{p}}\\ C_{p}=\frac{\pi }{2}\lambda_{p}r_{s}{}^{2}{\left(\frac{M_{1}}{M_{2}}\right)}^{p}{\left(\frac{2Z_{2}Z_{2}e^{2}}{r_{s}}\right)}^{2p} \end{array}\right.$$

where *θ* is the angle of incidence, $U_{b}$ (eV) is the surface binding energy of the workpiece material, *p* is a constant related to the incident ion, $\lambda_{p}$ is a function of *p*, $r_{s}$(mm) is the shielding radius, $M_{1}$, $Z_{1}$ (Da) is the mass of the incident ion and the number of protons, e denotes electronic charge [14].

Equation (8) shows that the removal rate of the pulsed ion beam is influenced by the incident ion energy, angle, beam density, and other stable factors during processing. Therefore, the removal function can be adjusted in real time by modifying the DC.

#### 2.1.2. Etching Model for Overall Etching Area

If the solution of the processing parameters of the unit surface element is extended to the entire sample processing surface, the duty cycle and removal height processing parameters of all surface elements need to be solved as a whole, so the duty cycle information of each surface element of the entire processing surface is recorded as the duty cycle vector DC; similarly, the removal height information is recorded as the removal height vector L composed of the removal height (l) of each unit cell, and the speed distribution information is recorded as the speed distribution vector V.

The following step involves establishing the overall etching region etching model. The velocity distribution vector is determined by the amount of removal. The speed of the machine tool axis usually ranges between 100 mm/min and 2400 mm/min, from which the velocity distribution vector can be obtained:

(9) $$V=\varphi \frac{2300}{L}$$

where φ is an adjustable proportionality parameter, which is used to compensate for velocity errors generated under different working conditions of the machine tool.

For the DC, the removal function $V_{e}$, and the processing time distribution vector T, the connection is given by Equation (10):

(10) $$\left\{\begin{array}{l} L=\left(T\cdot DC\right)*V_{e}\\ T=\frac{l}{V} \end{array}\right.$$

The set scanning mesh division length is represented by l. In Equation (10), this equation can be used to find the corresponding processing parameters for different target processing conditions by converting between DC, T, and L. It is also observed that L and T are linearly correlated when $V_{e}$ and DC are determined.

In the context of continuous ion beam figuring (IBF) during the machining of high-gradient aspheric components, the dwell time variation becomes excessively large in regions with significant material removal fluctuations. This scenario necessitates rapid acceleration and deceleration behaviors that exceed the capabilities of the machine tool’s motion axes within a constrained timeframe, resulting in a discrepancy between the actual and ideal operational speeds. Consequently, precise material removal cannot be achieved, despite the theoretical potential for nanometer-scale resolution in this machining method. Practical realization of this precision remains elusive, necessitating multiple iterative machining cycles, thereby rendering the process highly inefficient. In contrast, pulsed ion beam (PIB) technology discretizes the ion beam current through the control of pulse duty cycles, generating a pulse train with adjustable width and frequency. This approach mitigates the formation of additional removal layers, enhances machining convergence capabilities, and simultaneously reduces the demands on the machine tool’s dynamic performance.

### 2.2. Mask Plate-Based Graphic Transfer Method

To meet the requirements of integration, modularity, reliability, stability, and maintainability, as well as precise control, large-area atomic depth image transfer is needed for application in industrial automated production [15]. The cost of the device should also be taken into account. The mask plate used in this paper meets the requirements of industrial automated production due to its stable nature, high degree of etching shape freedom, good shape certainty, reusability, and affordability. The material properties and other factors are chosen to meet the processing requirements. The large-area graphics transfer method based on the mask plate is employed.

**Figure 3:**
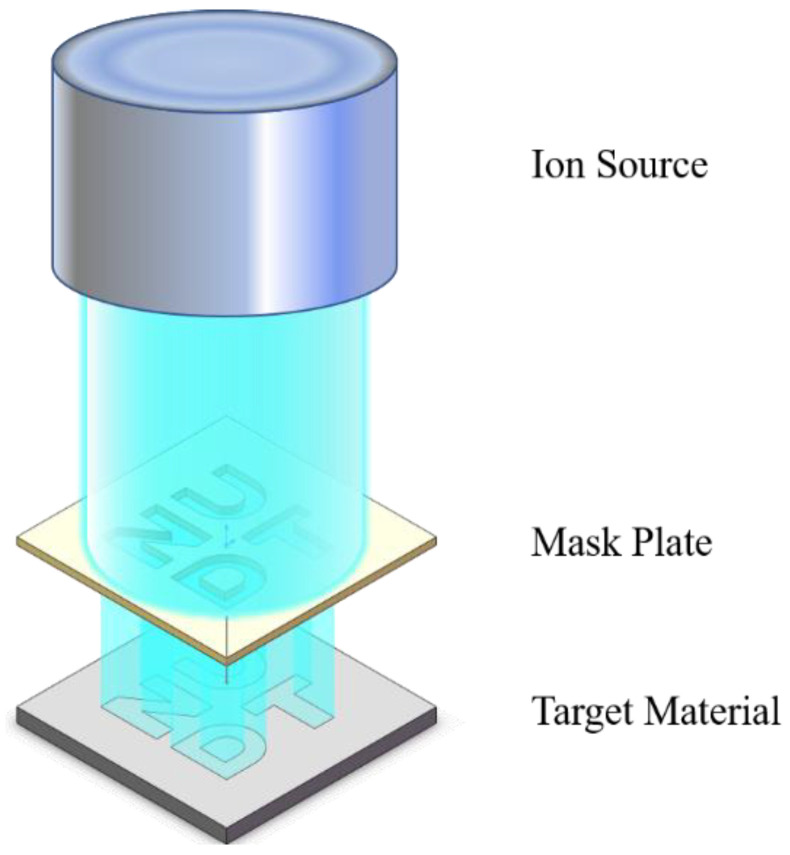
Mask plate-based graphic transfer method.

Figure 3 illustrates the specific atomic depth image transfer principle using the PIB process and mask plate. The ion source emits ion beams which, after passing through the mask plate, act on the target material. This process causes sputtering, resulting in the formation of graphics with a specific depth to complete the graphics transfer.

In the process of sputtering, the ion beam generates heat. Therefore, the mask plate material should be selected based on its ability to withstand the physical and chemical properties of the ion beam without changing and its heat resistance. Stainless steel was chosen as the mask plate production material due to its cost-effectiveness and ease of production.

## 3. Experimental Facilities and Methods

The research is based on the principle of mask plate image transfer. Firstly, the target pattern is designed based on the characteristics of the quartz sample. Then, the experimental processing device is designed and prepared. The processing device conceptual diagram and physical figure are shown in Figure 3. The device substrate is machined using CNC with a machining precision error of ± 0.05 mm. The mask plate is prepared using an ultra-high-precision laser machining method with a machining error of ±20 μm.

For this study, quartz samples with specifications of 10 × 10 × 1 (mm) are selected. According to the Geometrical Product Specification (GPS) [16], the arithmetic mean deviation surface roughness ($R_{a}$) of the samples is less than 0.1 nm when the sampling length is 0.08 mm, indicating that the samples have smooth surfaces.

**Figure 4:**
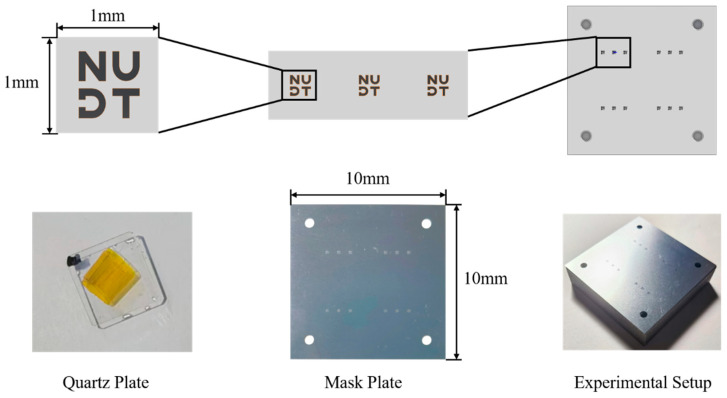
Experimental setup.

This paper aims to explore and verify the actual role of the mask plate-based image transfer method and the relationship between T of the target etching region and the target removal height distribution vector L under a fixed duty cycle vector. To achieve this, the pulsed-type ion beam processing equipment IBF-700 developed in-house by the laboratory was used to process a specific region of two quartz samples. The processing equipment is shown in Figure 4, and the processing time vectors of the three processing regions of sample A were recorded based on the theoretical model in Section 2. $T_{A}=[\begin{array}{ccc} 15 & 22.5 & 30] \end{array}$, the vector $T_{B}=\left[\begin{array}{cc} \begin{array}{cc} 37.5 & 45 \end{array} & 52.5 \end{array}\right]$ represents the machining time for sample B. Other specific machining parameters used in the experiment are detailed in Table 1. Following machining, the etched area of each machined region was measured using a white light interferometer, specifically the Zygo Verifire Asphere (Zygo Corporation, Middlefield, OH, USA).

## 4. Results

### 4.1. Preliminary Analysis of Experimental Results and Data Processing

**Figure 5:**
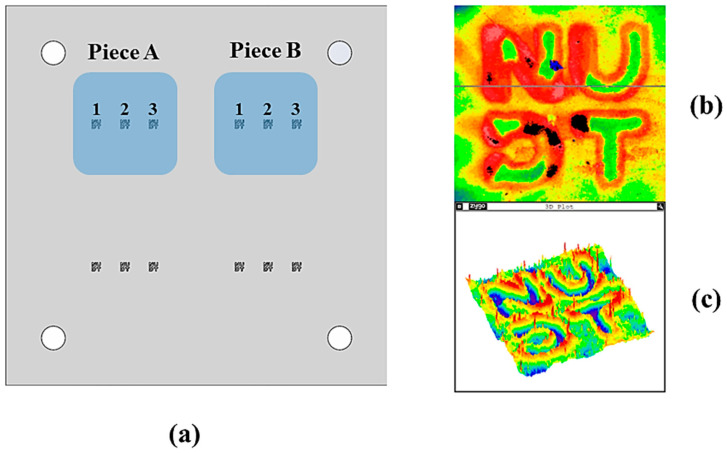
(**a**) Schematic diagram of the machining patterns and parameters of the sample; (**b**) Poorly etched area; (**c**) Better-etched area.

The machining quality of the processed etching area was observed and analyzed in the test. The regions with better processing quality were identified as region 2 of sample A and regions 1 and 2 of sample B, as shown in Figure 5a. Figure 5b,c show the imaging images of the better-etched area and the poorer-etched area, respectively. It has been observed that well-processed patterns are characterized by clear and relatively uniform depths of the etched grooves, whereas poorly processed patterns exhibit extensive deviations in groove depths, non-uniformity, and damage to the etched surfaces.

Regarding distribution, the well-defined patterns are primarily situated to the left of the sample’s center and in the section with a longer sputtering time. The processing quality is mainly influenced by the positioning or matching error of the processing device and the sputtering time of the ion beam, as determined from the analysis of the processing device and ion beam processing. The processing device’s fitting error primarily affects the central point of the pulsed ion beam’s action. This is because the energy deposition of the beam follows a Gaussian distribution, and any shift in the central point will result in uneven energy deposition across the processed area. This, in turn, can cause some particles to remain attached to the surface, resulting in shallow etching depth and the appearance of faults in the processed area [17], as shown in Figure 5b. The time taken for ion beam sputtering also affects the degree of incomplete detachment of particles from the target etching area by influencing the energy deposition. The error in the deposited energy accumulates with an increase in processing time, which affects the relative error in the depth of etching in the processed area, as well as the uniformity of the etching area and etching.

### 4.2. Measurements and Calculations of Parameter Indicators in the Processing Area

The analysis of the etching depth focuses on the higher-quality etched area after processing. Figure 5b shows that the measured data contain spike noise, Gaussian noise, and prominent outliers. Therefore, data processing is carried out first.

The median filter is a statistical ordering filter, which belongs to the nonlinear filter. Its principle is: that the gray values of all pixels in the neighborhood of each point in the input image are sorted by size, and its median value is selected as the gray value of the corresponding point in the new image, so as to achieve the effect of image denoising.

Gaussian filter is a linear smoothing filter widely used in the field of image processing, which is mainly used to reduce the image noise and details and realize the smoothing effect of the image. For a two-dimensional image, the expression of the Gaussian neighborhood averaging filter is:

(11) $$g(x,y)=e^{-\frac{{\left(x-a\right)}^{2}+{\left(y-b\right)}^{2}}{2\sigma^{2}}}$$

where (*a*, *b*) is the position of the Gaussian peak, which is generally set to coincide with the center of the filter, at once $a=\frac{N-1}{2},b=\frac{M-1}{2}$, where (*M*, *N*) is the filter size, and σ is the standardized variance. For some image processing software or libraries, it is usually possible to specify the σ value directly, and the kernel size will be calculated automatically.

**Figure 6:**
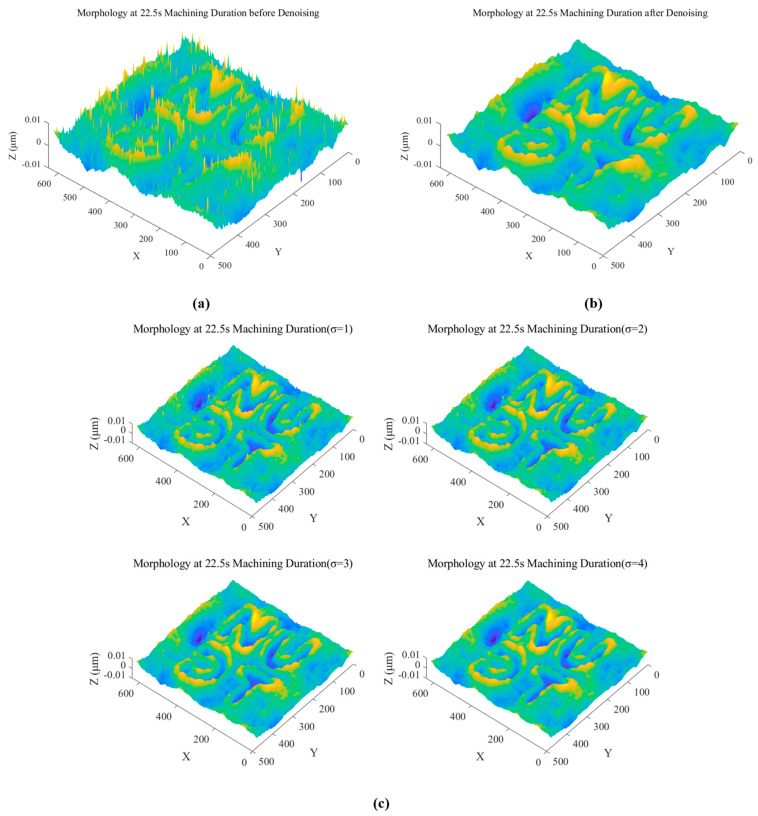
(**a**) Before denoising; (**b**) After denoising; (**c**) Denoising effect under different σ.

As median filtering is not effective for Gaussian noise filtering [18,19], Gaussian filtering is a better option and has been applied in several cases of wave imaging denoising [18]. A comparison was conducted between the noise reduction effect and the different σ parameters; for the noise reduction of the imaging data, both median filtering with a 5 × 5 window and Gaussian filtering (σ=4) were successively used. The effect diagrams before and after processing are shown in Figure 6a,b, denoising effect under different σ are shown in Figure 6c.

**Figure 7:**
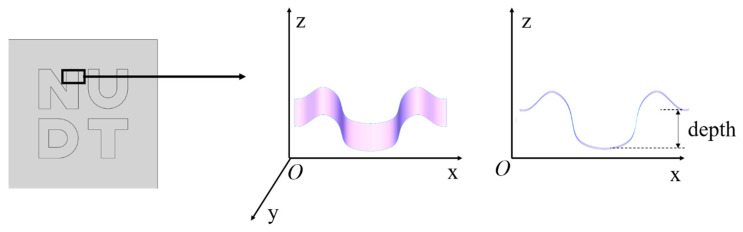
Depth of etch.

Figure 7 shows the process for estimating etching depth in rectangular areas with a uniform selected etching depth and etching edge on both sides of the flat. The letter etching pattern differs for each processing area. The depth of the rectangular area is estimated through the step section.

**Figure 8:**
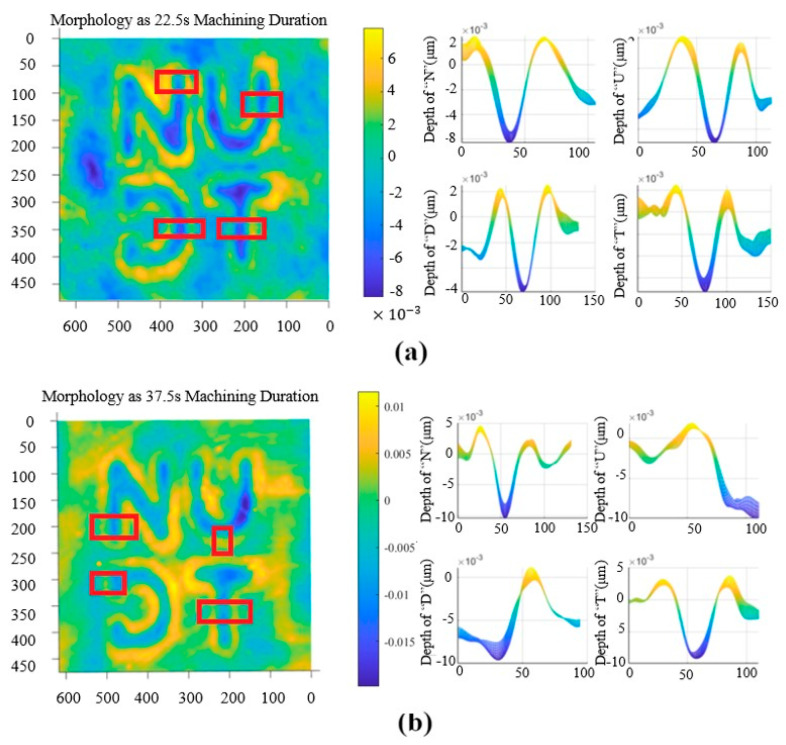

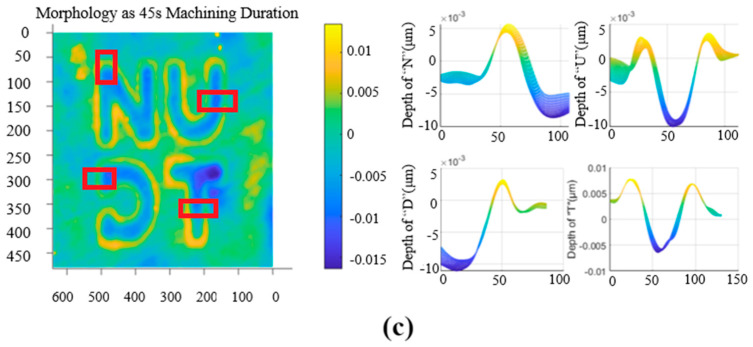
The shape of the processed area and X–Z section of the groove at different sputtering times: (**a**) 22.5 s; (**b**) 37.5 s; (**c**) 45 s.

In the methodology for measuring the roughness of the etched areas, a random selection of four distinct regions, each measuring 0.08 mm by 0.08 mm, is conducted at the center of the etching grooves within each small pattern. Following the measurement and calculation of these areas, the average value is determined, which serves as the average roughness of the machined area $R_{a}$ for the etched region under the specific sputtering time. The results of the measurement are presented in Table 2 and Figure 8.

## 5. Discussion

The experimental results are first analyzed qualitatively. For the cross-section morphology step, as the etching depth of the processed area increases, sputtered atoms gradually accumulate on both sides of the groove bottom due to their difficulty in escaping. This results in a Gaussian distribution of the etching area, which is consistent with the basic law of pulsed ion beam etching [14,20]. Additionally, the effect of material redeposition forms a ring wall at the edge of the etching area, which is consistent with the characteristic law verified by existing research [21].

**Figure 9:**
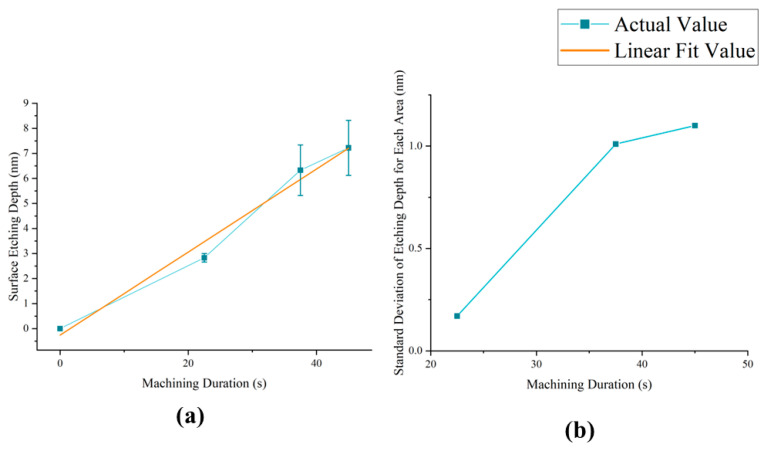
(**a**) The folded line and linearly fitted straight line for the mean sputtering time-etching depth. (**b**) The folded line for the mean sputtering time-etching area depth.

Quantitative analyses were conducted next. The results obtained from a linear fit to the average sputtering time of the etched region are presented in Figure 9a.

The fitted linear equation is shown as follows:

(12) L(t)=0.1659t−0.26

The fitted adjusted R-squared is 0.97207, indicating a strong linear positive correlation between the average sputtering time of the etched area and the etching depth, assuming that other conditions are determined. This conclusion is consistent with the findings in Section 2.1.2, suggesting a good fit of the model with the experimental results. It can be inferred that the etching rate of the PIB is approximately 12 nm/min when using $\mathrm{Si}O_{2}$ as the target material.

Figure 9b shows the standard deviation of the etching depth for each etching region at different sputtering times. The standard deviation of the depth between the etching regions increases gradually with the growth of sputtering time. This reflects the influence of device coordination error and the cumulative error of the deposition energy generated by ion beam sputtering. These findings are consistent with the conclusions obtained from the qualitative analysis.

The most commonly used nanofabrication processing methods are atomic layer etching (ALE) in traditional chemical method etching and focused ion beam (FIB) and reactive ion etching (RIE) in ACSM. When considering large-area atomic depth image transfer in industrial automated production, the main factors to consider are removal resolution, etching rate, and process complexity. Although FIB has a high removal resolution, it is not suitable for large-area etching due to the high number of repetitions and low etching rate of $\mathrm{Si}O_{2}$ etching by FIB without the aid of auxiliary processes such as wet etching. Therefore, it is not suitable for large-area etching [22]. Chemical etching can improve the etching rate to a certain extent [23], but using it as an auxiliary process increases the complexity of the overall process and poses a greater obstacle to industrial automation of production. Therefore, it is not suitable for large-area image transfer as an etching method. A comparative analysis was conducted on the RIE, ALE, and PIB methods. Given that the processing area of the three methods can be subjected to atomic-scale processing, this thesis employs the depth change of the etched area per unit time as the metric for measuring and comparing the etching speeds. Let the etching depth be denoted by $l_{e}$, the corresponding etching time by $t_{e}$, and the etching speed by $v_{e}$. Then, we have:

(13) $$v_{e}=\frac{l_{e}}{t_{e}}$$

Subsequently, the approximate etching rate for each of the three etching methods is as follows: 35–40 nm/min [24], 0.25 nm/min [25], and 10 nm/min. The etching rates of RIE and PIB are much higher than that of ALE. However, the removal resolution of RIE is insufficient to meet the accuracy needs of atomic depth image transfer. Similarly, PIB can only meet the accuracy needs of sub-micron accuracy by adjusting the removal resolution, but not the atomic depth of image transfer accuracy. PIB can achieve the required image transfer accuracy through adjustments. However, the removal resolution of RIE is low, which can only meet the demand for sub-micron accuracy and cannot achieve the accuracy required for atomic depth image transfer. On the other hand, PIB can achieve an equivalent resolution (defined as the resolution obtained by averaging the measurements after processing with multiple pulses until the step height can be detected) of 6.4 × 10^−4^ nm at the pulse width of 0.01 ms [14]. In summary, for the atomic depth image transfer process, PIB can fully meet the requirements of industrial automated production in terms of efficiency and accuracy and has better properties than other etching methods.

The preliminary results of the mask plate-based graphic transfer method have demonstrated the feasibility of image transfer based on the designed processing device. Furthermore, the linear law of etching depth versus time for SiO_2_ processed at nanoscale depth has been obtained, indicating that the experimental verification is in good conformity with the theoretical model. Following the completion of the aforementioned work, the method can be further improved in the next step, including the following aspects: (1) Further increase or reduce the processing time, and processing area, and explore the feasible conditions and maximum accuracy of the method under more extreme processing conditions. (2) Optimize the processing device, reducing errors caused by the device and improving processing accuracy, while simplifying the device preparation. The process and structure of the device will be improved to reduce the errors caused by the device, further improve the processing accuracy, and simplify the preparation process of the device. Additionally, further theoretical analysis of the conditions for obtaining high-quality processing results will be conducted, with the objective of improving the stability of the processing results and the etching quality.

## 6. Conclusions

This paper proposes a method for achieving large-area atomic depth pattern transfer using a pulsed ion beam and metal mask plate. This paper analyses the atom-level control of the pulsed ion beam etching process and mask plate etching technology through theoretical models and experimental verification. It obtains the etching effect of each region of the target pattern under different sputtering time conditions and calculates key parameters such as the etching rate. The proposed etching model and atomic depth pattern transfer method are confirmed to be valid. Compared to traditional nanofabrication technology, this method offers higher removal resolution, faster etching rates, and lower process complexity in automated industrial manufacturing, providing clear advantages. The results demonstrate that the method can achieve precise control of etching depth with a linear relationship to sputtering time while maintaining sub-nanometer average surface roughness at an etching rate of approximately 10 nm/min for quartz materials on a second timescale. However, the standard deviation of the etching depth increases as the sputtering time extends, indicating the impact of system fit error and cumulative error of the ion beam sputtering energy. A comparison of PIB with existing nanofabrication methods shows that, for the atomic depth image transfer process, PIB can meet the requirements of industrial automated production in terms of efficiency and accuracy, and it has superior properties compared to other etching methods. In conclusion, this study confirms the feasibility of using pulsed ion beam-based atomic depth image transfer technology for large-area optical materials in industrial production. Additionally, the study improves the theoretical model of the PIB technology and expands its application in the field of industrial automation.

## Figures and Tables

**Table 1:** Experimental parameters for pulsed ion beam removal.

Parameters	Value	Parameters	Value
Ion type	Ar+	Pulse duty cycle	20%
Screen grid voltage	800 V	Pulse frequency	1 Hz
Acceleration grid voltage	−120 V	Angle of incidence	90°
Deceleration grid voltage	0 V	Grid aperture	15 mm
Distance between mask and substrate	0.5 mm		

**Table 2:** The results of measuring the etching depth at different processing times.

**Sputtering Time t (s)**	**Depth of Etching Area “N” (nm)**	**Depth of Etching Area “U” (nm)**	**Depth of Etching Area “D” (nm)**	**Depth of Etching Area “T” (nm)**
22.5	2.77	2.62	2.97	2.97
37.5	7.27	6.65	4.89	6.50
45	5.75	7.68	8.33	7.12
**Average Depth of Etched Area (nm)**		**The Standard Deviation of the Depth of the Etched Area**	**Average Roughness of Machined Area (nm)**	
2.83		0.17	0.33087	
6.33		1.01	0.57698	
7.22		1.10	0.43101	

## Data Availability

Data are contained within the article.

## Abbreviations

Y(E)	The surface sputtering yield of ion beam (atoms/ion)
$U_{s}$	Surface binding energy per atom (eV)
$M_{1}$	The mass of the incident ion (Da)
$M_{2}$	The atomic mass of the target material (Da)
$S_{n}$	Nuclear-stopping cross section ($cm^{2}$)
$S_{n}^{TF}$	Nuclear-stopping power based on the Thomas–Fermi potential (eV/Å)
$Z_{1}$	Atomic numbers of the incident ions
$Z_{2}$	Atomic numbers of the target atoms
ε	Normalized energy
$\varepsilon_{o}$	Incident ion energy (eV)
E	Energy distribution pattern of the incident ions
μ	Scattering width of the incident ion energy
σ	Scattering width parallel to the incidence direction
$E_{o}$	The pulsed ion beam’s outgoing energy (eV)
f	Frequency (Hz)
DC	Duty cycle
t	Sputtering time (s)
I	Intensity of the ion beam (mA)
$J_{s}$	The current density of the single-aperture beam (mA)
V	The total acceleration voltage (V)
$L_{g}$	The distance between the screen grid and the acceleration grid (mm)
$M_{i}$	The mass of the ions (Da)
e	The charge of the electric charge
$\varepsilon_{0}$	The electric constant
$d_{s}$	The diameter of the beam aperture (mm)
dΩ	The unit grid cell of the workpiece
η	The proportionality constant
$U_{b}$	The surface binding energy of the workpiece material (eV)
p	Constant related to the incident ion
$\lambda_{p}$	Function of p
$r_{s}$	Shielding radius (mm)
θ	The surface binding energy of the workpiece material (degrees)
DC	Duty cycle vector
L	Removal height vector
l	The removal height of each unit cell
V	Speed distribution vector
φ	Adjustable proportionality parameter
T	Processing time distribution vector
$V_{e}$	Removal function
